# Impact of practitioner's training in the management of alcohol dependence: a quasi-experimental 18-month follow-up study

**DOI:** 10.1186/1747-597X-1-18

**Published:** 2006-07-14

**Authors:** Laurent Malet, Michel Reynaud, Pierre-Michel Llorca, Bruno Falissard

**Affiliations:** 1 CHU Clermont-Ferrand, Clermont-Ferrand, F-63000, France; 2 Inserm, U669, Paris, F-75014, France; 3 Univ Paris-sud 11, Le Kremlin Bicêtre, F-94000, France; 4 Univ Paris 5, Paris, F-75015, France; 5 AP-HP, Villejuif, F-94804, France

## Abstract

**Background:**

In many European countries, medical education on alcohol remains inadequate in terms of both quantity and quality. The expansion of GP training and care protocols would improve the management and outcome of alcoholic patients. Our purpose was to assess the impact of a multifaceted intervention by trained GPs in the management of alcohol-dependent patients.

**Results and discussion:**

Trained GPs proved better i) in the attempt at abstinence, with 67% patients becoming sober *vs*. 47% in a comparison sample and ii) in repeat attempt at abstinence in the event of relapse, with an average 2.99 *vs*. 1.31 attempts per patient. There were no differences in terms of i) relapses, which involved about three in four patients, and ii) prolonged abstinence, which averaged two months. Overall, patients managed by trained GPs remained abstinent 103 days during the 18-month follow-up period vs 68 days for the comparison sample (p = 0.016).

**Methods:**

This 18-month follow-up study had a quasi-experimental design with 24 volunteer trained GPs and a comparison sample of a representative sample of 24 GPs. All GPs included their own already existing DSM-IV alcohol-dependent patients. Patients with depression or anxiety comorbidities were included. Participants were 126 patients in the trained sample and 122 in the comparison sample. The two patient samples were evenly-balanced, averaging 47 years old and 80% males. In the trained sample, consultations were scheduled and management (medication, biological workup) was protocolized, whereas the comparison sample representing standard practice had no obligations.

**Conclusion:**

Medical education can sharply improve the management of alcohol-dependent patients and short-term outcome. Trained GPs lead more patients to attempt abstinence and more often than in standard practice. However, a strict medical approach remains limited in the maintenance of medium-term abstinence (over two months), providing a strong argument for multidisciplinary management of alcohol-dependent patients.

## Background

General practice represents the primary care system in France. Three quarters of the French population consult a GP at least once every year, and 92% stay nested within their attending physician [1].

Twenty percent of GP's patients present with alcohol use disorders (5% dependence) [2]. Although these alcohol-related problems are relatively common, GPs are still finding it difficult to identify and manage these patients [3]. GPs commitment or ability may be hampered by individual factors such as knowledge levels, skills, attitudes, beliefs and expectations. These factors can be improved by education and training [4,5]. However, initial medical training on alcohol misuse varies between French universities, and represents only 4–10 hours of instruction [6].

**Figure 1:**
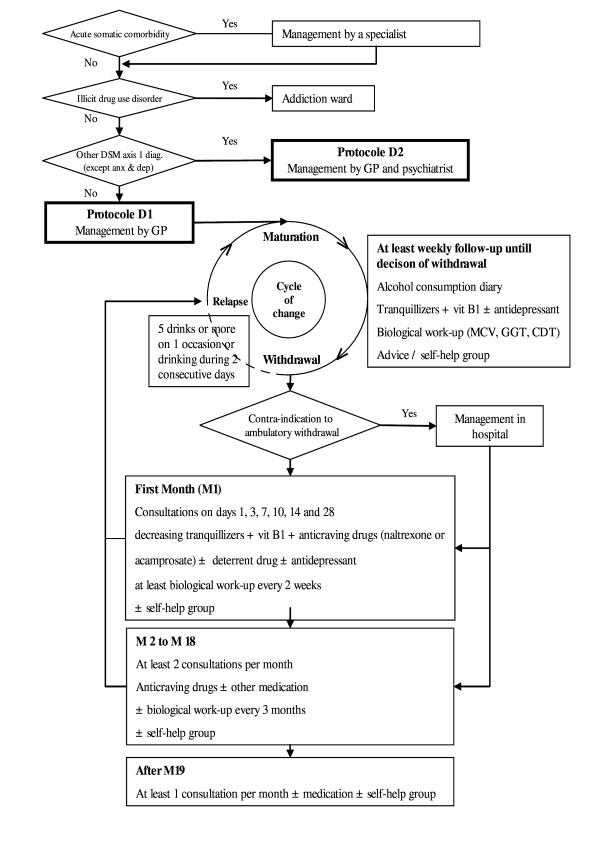
Summary of follow-up by GP alone (protocol D1) in the trained sample. A minimum consultation frequency is scheduled according to patients' state of change. Prescriptions (medications, biological work-up) are protocolized. Contra-indications to ambulatory withdrawal are based on the recommendations of the French Society of Alcohology [9, 10].

The centre of France ranks among the leading French regions in terms of incidence of alcohol use disorders [7]. Local training initiatives have been set up for volunteer GPs covering alcohol misuse (especially alcohol dependence) and healthcare protocols. In 2001, these practitioners were integrated into an experimental healthcare network approved by the French Ministry of Health [8]. They were given university training with field-work instruction (available in every French university) followed by ongoing training comprised of role-playing and simulation exercises. Figure 1 gives a brief illustration of the healthcare protocol (based on the recommendations of the French Society of Alcohology [9,10]) corresponding to over 80% of the patients included in the network. This protocol mainly gives a framework for follow-up. It defines a minimal frequency of consultation according to the patient's state of change. The systematic use of follow-up tools such as self-questionnaires and biological work-up is reported to improve patient motivation. Medical files are managed via a networked database for patient follow-up that gives the stage of the protocol and also manages payment of the practitioners. Remunerations are based on an all-inclusive fee taking into account this time-intensive management strategy. The functioning of the network is exhaustively described in the Official Journal of the French Republic, which can be found at [11].

The aim of this study was to assess the impact of this practitioner training plus healthcare protocol strategy on outcome in alcohol-dependent patients versus a comparative sample during an 18-month follow-up period. Each GP included any of his or her existing eligible patients according to the quasi-experimental design of the study.

## Results and discussion

### Practitioners and patients

All volunteer practitioners in the trained sample were males and their average experience as a GP was 14.3 years [SD 5.1]. In the random comparison sample, the GPs (70% were males) had an average 17.6 years [SD 6.3] of experience and had received an average 10 hours of tutoring on alcohol misuse during their medical studies.

126 patients were included in the trained GPs sample, i.e. 5.3 [SD 4.9] patients per GP, and 122 patients were included in comparison sample, i.e. 5.1 [SD 4.4] patients per GP.

The two patient samples were very similar in terms of socio-demographic and clinical data (Table 1). The male-to-female gender ratio was 5/1, with an average age of 47 years old. These characteristics are usual in alcohol misuse studies incorporating clinical samples [12]. There were more physical dependents (92 *vs*. 84%) and significantly (Fisher's exact p = 0.04) longer-standing alcohol problems in the trained sample. There was a very high incidence of psychiatric comorbidities in both the trained and the comparison sample (anxiety 79% and 84%, depression 55% and 57%, respectively: Fisher's exact p = 0.24 and Fisher's exact p = 0.77) which is in agreement with the highest prevalence reported in the literature [13-15].

**Table 1:** Sociodemographic and clinical data. All percentages were tested using Fisher's exact and continuous variables were tested using Mann-Whitney tests

	**Study population**		**Patients attempting abstinence**		**Relapsing patients**	
	
	Trained sample	Comparison sample	Trained sample	Comparison sample	Trained sample	Comparison sample
	n = 126	n = 122	n = 85	n = 57	n = 74	n = 38
**Gender (% of men)**	80%	83%	82.4%	86%	86.5%	81.3%
**Age (mean [SD])**	46.9 [9.1]	47.8 [9.1]	47 [8.6]	46. [8.8]	46.5 [8.9]	46.9 [8.]
**Physical dependence**	92.1%	84.4%	90.6%	91.2%	92.2%	94.6%
**Severity of dependence**						
moderate (3 or 4 items)	33%	41%	34%	32%	33%	32.5%
mild (5 or 6 items)	40%	39,5%	41%	45%	39%	43%
severe (7, 8 or 9 items)	27%	19,5%	25%	23%	28%	24.5%
**Duration of alcohol misuse**						
less than 2 years	5%	**6%**	5%	5%	8%	6%
3 to 5 years	11%	**15%**	10%	17.5%	16%	11%
6 to 10 years	8%	**19%**	10%	17.5%	16%	9.5%
more than 10 years	76%	60%*	75%	60%	60%	73.5%

* p < 0.05

The results are based on a total of 1,867 consultations in the trained sample, i.e. 15.5 [SD 13.8] per patient, and 883 consultations in the comparison sample, i.e. 7.2 [SD 4.5], over an 18-month period. The incidence of a management strategy was higher in the trained sample, fitting with the protocol.

### Lost to follow-up and deceased

Patients lost to follow-up were those patients included and then never seen during the 18-month follow-up period. Ten patients were lost to follow-up in the trained sample (8%) and 3 (2%) in the comparison sample (Fisher's exact p = 0.09). Loss to follow-up at one year varies between 15% and 30% in general practice studies [16]. Our very low rate of loss to follow-up at 18 months is probably due to the quasi-experimental design of the study and confirms the patients' loyalty to their attending practitioner. In both samples patients are nested within their practitioner like 92% of the French population.

Three patients (2%) in the trained sample and 9 patients (7%) in the comparison sample died during the study, all due to alcohol-related complications (Fisher's exact p = 0.12). Mortality was consistent with previously reported figures for a cohort follow-up study of alcohol misuse [17].

### Attempts at abstinence and relapses

Attempts at abstinence were more frequent in the trained sample: 67% of patients stop drinking compared to 46% of patients in the comparison sample (Fisher's exact p < 0.0001). At the last recorded observation, 23% of patients in the trained sample were abstinent compared to 17% in the comparison sample (Fisher's exact p = 0.36). However, relapses were more frequent in the trained sample (87 *vs*. 67%, Fisher's exact p = 0.003). Longest period of abstinence was significantly different between the two samples but appeared to be relatively short and very close in both samples: 68.1 days [SD 9.9] in the trained sample vs 61.7 days [SD 9.7] in the comparison sample (Mann-Whitney chi-square = 6.32, df = 1, p = 0.01). There were no differences in any socio-demographic characteristics or alcohol consumption profiles between study samples, subsets of patients attempting abstinence or the subset of relapsing patients (Table 1).

### Repeated attempts at abstinence and relapses

Average frequency of attempt at abstinence and number of relapses per patient were twofold higher in trained sample patients than comparison sample patients (2.99 *vs*. 1.35 for attempts and 3.23 vs. 1.21 for relapses). In addition to higher initiation rates for attempt at abstinence, the main between-sample difference concerned the ability of the trained sample to repeat abstinence attempts, leading to a much longer cumulative duration of abstinence: 102.9 days [SD 13.5] in the trained sample compared to 68.4 days [SD 10.6] in the comparison sample (Mann-Whitney chi-square = 9.63, df = 1, p = 0.002). Trained sample patients remained abstinent for an average 36% of the follow-up period, compared to only 16% for the comparison sample (Fishers' exact p = 0.016).

**Figure 2:**
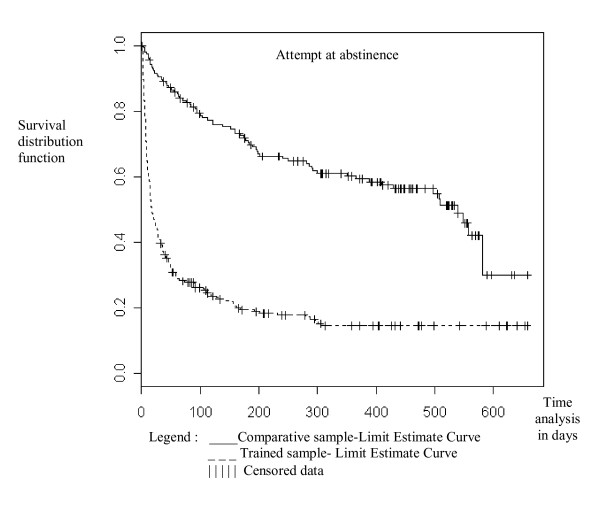
Kaplan-Meier survival estimates attempt at abstinence, by sample. The abscissa represents follow-up duration in days. Each step is an attempt. Data are censored if attempt at abstinence does not occur. Difference between the two samples is significant in a Cox regression test with and without adjustment.

**Figure 3:**
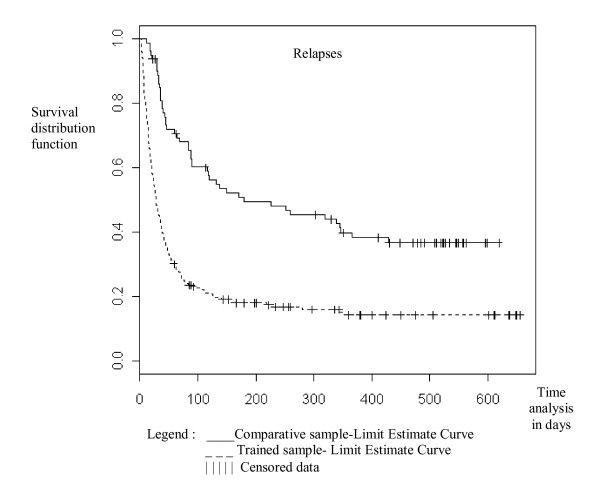
Kaplan-Meier survival estimates relapses, by sample. The abscissa represents follow-up duration in days. Each step is a relapse. Data are censored if relapse does not occur. There is no difference between the two samples in a Cox regression test when adjusted.

These different between sample-patterns were represented graphically, showing survival analysis and repeat attempt at abstinence patterns in the trained sample (Figure 2) and the high relapse rate during the first two or three months after drinking cessation in both samples (Figure 3). Differences are significant in a Cox model for multiple events (for attempts at abstinence: Wald chi-square = 14, df = 1, Cox p < 0.0001; for relapses: Wald chi-square = 37.2, df = 1, Cox p < 0.0001) and were confirmed with the log-rank test in both cases when the model included each patient only once, at first attempt at abstinence or first relapse.

A multivariate Cox model of multiple events was run to identify whether between-sample differences persisted following adjustment for other covariates:

- attempt at abstinence remained related to a sample effect (Wald chi-square = 11.65, df = 1, Cox p < 0.001), but was also related to frequency of consultations (Wald chi-square = 23.78, df = 1, Cox p < 0.0001) and the prescription of benzodiazepines (Wald chi-square = 4.49, df = 1, Cox p = 0.03), which were frequently prescribed in both sample and are considered standard practice for any GP in the early stages of withdrawal for these mainly physically-dependent patients [18].

- for relapses, the adjustments cancelled out the between-sample difference (Wald chi-square = 2.04, df = 1, Cox p = 0.15). The occurrence of relapse appeared to only be related to frequency of consultations (Wald chi-square = 15.80, df = 1, Cox p < 0.0001) and onset of anxiety disorder (Wald chi-square = 5.52, df = 1, Cox p = 0.02). This link between alcohol consumption and anxiety has been described previously [19,20].

- for both attempts at abstinence and relapses, we observed no significant differences related to age, gender, DSM-IV severity, duration of alcohol misuse, occurrence of a major depressive episode, or prescription of anticraving drugs or antidepressants.

Trained GPs clearly targeted alcohol dependence. They knew how to address the alcohol issue and accompany patients towards drinking cessation. They knew to put relapses into perspective, considering them as stages in the process rather than perceiving them as failures. Training enhanced their ability to motivate patients and to initiate and repeat attempts at abstinence. It is also likely that the frequent successive consultations (twofold more consultations scheduled than in the comparison sample) enabled rapid screening of relapses, and thus earlier and therefore more efficient interventions. On a subjective level, regular meetings between GPs in the network provided a forum for discussion of individual cases and exchange of experience, and probably also enabled the trained GPs to maintain the necessary resolve to keep working on high-relapse patients.

### Study limitations

The trained sample was formed from a local, experimental healthcare initiative authorised by the French Ministry of Health which controlled the adherence to the protocol. Volunteer practitioners settled the comparative experimentalist design of the study, and thus precluding randomization. The comparison sample comprised the same number of practitioners, giving a representative sample of GPs in the same geographical area. Even if physicians were the primary sampling unit, the evenly-balanced and clinically similar samples of patients provided a good basis for comparison. The study remained pragmatic, using the regular practitioner in order to maintain any established patient-doctor relationships, which overall gives quite good generalizability.

This intervention combines GP training with application of a follow-up protocol. Both strategies are easily reproducible despite that self selected GPs had probably already an interest or a sensitisation about alcohol problems. The ongoing training through role-playing and the experience exchange forum (particularly focusing on the more difficult cases) is more innovative and partly informal. It is therefore difficult to differentiate the individual effects of each component in this complex intervention strategy. Nevertheless, it is probable that the more regular consultations scheduled by the protocol promoted patient maturation, and would thus explain the greater proportion of attempts at abstinence. Similarly, the protocol meant that relapses were screened earlier. It is likely that the alcohol misuse training improved the GPs skills with alcohol-dependent patients, in particular by enabling them to perceive relapses as stages in the process rather than failures, and increasing their ability to repeat attempt at abstinence if necessary.

## Conclusion

This study confirms the crucial role of the GP in the management of alcohol dependence. Given patients' loyalty to their practitioners and the very low lost to follow-up rate in standard general practice, alcohol consumption has to be a major issue for any GP. Medical training improves the short-term outcome (few weeks) of alcohol-dependent patients, i.e. 50% higher attempt at abstinence rates and twofold longer cumulated abstinence duration than standard practice.

The quantitative aspect of medical management seems important. Frequent and regular visits (as planned in the protocol according to the patients' state of change) give time for maturation and enforce the patient-practitioner alliance. Obviously, financial compensation, even if not an incentive, remains essential for this time-intensive management strategy.

The maintenance of medium or long-term abstinence (over few months) does not appear to depend on any kind of medical management. In both samples, sustained sobriety appeared to be relatively short and did not exceed two months. This result highlights the limits of a strictly medical approach which has to be pre-eminent in the early stages of abstinence attempt. Maintenance of long-term abstinence would therefore appear to be far more dependent on a multidisciplinary management strategy [21-25]. Prospective orientations for the network include involving different professionals, collaborating with social workers and psychologists, and strengthening the contribution of self-help groups [26,27]. Given the lack of established guidelines and references, the protocolization of interventions for all professionals involved (who, when, how?) remains a challenge.

## Methods

This aim of this open, prospective, two-sample study was to compare management of alcohol-dependent patients between trained and untrained practitioners. The comparative, quasi-experimental design was devised to compare an experimental sample of volunteer trained GPs with a comparison sample [28]. Existing patients were included over 9 consecutive months by their own practitioner (whether belonging to the trained network or not) as and when they became eligible. Patients were therefore not randomized. Follow-up period was 18 months for each patient. The protocol was approved by the appropriate French ethics committees, and all participating patients signed an informed consent form.

### Practitioners and follow-up

The trained sample comprised 24 volunteer practitioners. Their follow-up fitted protocol D1 (patients with no psychiatric comorbidities other than anxiety or depression, and management by GP alone), as summarised in figure 1. This protocol is essentially based on a regular consultation schedule that is lightened as the patient progresses in a cycle of change through maturation, attempt at abstinence, medium-term sustained abstinence (2 months), and eventually long-term remission. A once-weekly consultation was scheduled during the maturation phase, then two to three consultations per week over the next three weeks when attempting at abstinence, and so on. Further consultations could be scheduled if the GP deemed it necessary. The idea behind training the GPs was intended to facilitate and accelerate patient maturation, both from the outset as well in response to relapses. The tools used (consumption diary, biological work-ups, etc.) were designed to act as follow-up indicators or to set goals, or even to be used as motivational tool. The prescription of medical drugs remained the practitioner's choice. Only prescriptions of thiamine and benzodiazepines (short-term) were made obligatory during withdrawal, in compliance with the recommendations. It was systematically recommended that anti-craving drugs were prescribed.

For the comparison sample, 24 GPs were chosen at random from the official public list of physicians to provide a representative sample (according to age and sex) of GPs in the same geographical area. GPs forming the comparison sample attended sessions in order to become familiar with the Mini International Neuropsychiatric Interview (MINI) for diagnosing DSM-IV dependence, anxiety and mood disorders [29,30]. Consultation frequency in the comparison sample was open-ended.

### Patients

All patients included in the comparison sample were aged over 18 and met DSM-IV criteria for alcohol dependence. General exclusion criteria were: patients for whom alcohol dependence was not the main diagnosis on axis 1 of DSM-IV (mental retardation, schizophrenia or other psychotic disorder, bipolar mood disorder), any other addiction (except tobacco), and severe personality disorders (in particular psychopathic and borderline patients). Subjects with anxiety or depression were not, however, excluded. These criteria corresponded to patients attending protocol stage D1 in the trained sample.

### Measures

Patients were described according to the usual sociodemographic data. At baseline, severity of dependence was evaluated by summation of DSM-IV criteria, and simplified by breakdown into 3 categories: moderate, mild or severe [31]. DSM-IV criteria were also used to assess physical dependence. Duration of alcohol misuse was considered as an indicator of severity.

Given that psychiatric comorbidities can be predictors of outcome in alcohol dependence [19,32], anxiety and mood disorders were accurately evaluated at each consultation using the hospital anxiety and depression scale (HADS) [33] for the trained sample, and the Mini International Neuropsychiatric Interview (MINI) for the comparison sample [29]. Self-questionnaires were avoided in the comparison sample due to potential bias associated with maturation.

All medical prescriptions, particularly of benzodiazepines, antidepressants and anticraving drugs (acamprosate or naltrexone), were recorded.

Abstinence was the main outcome measure. Patients were asked to state their alcohol consumption using a detailed consumption diary or the consumption items in the AUDIT questionnaire [34]. The highly-specific diary collection in the trained sample can be considered as a motivational tool, and was not used in the comparison sample as a potential bias. All practitioners had to indicate their impressions concerning a false declaration of abstinence, and detail their arguments: patients obviously intoxicated, results of biological work-up, information from the family circle, etc.

### Analysis

Both samples were compared based on sociodemographic and clinical data.

Patients who died or who were lost to follow-up were compared between the twosamples, as were percentages of attempts at abstinence and relapses and averages of longest period of abstinence and cumulated abstinences.

We conducted a survival analysis based upon Cox regression as a substitute for logistic regression. This method is optimal when the dependent variable (attempt at abstinence or relapse) is a binary event, and where is also information on the length of time to the event (which is the pattern of the study) or this may be censored if the event does not occur [35]. Besides, we used more sophisticated techniques to account for the clustering of patients within physician and obtained results that were essentially the same as from simpler analyses assuming independence. Thus, we present the simpler results only.

Two types of survival curves were plotted using the Kaplan-Meier method: curves where each patient could figure several times (if relapsing then attempting at abstinence again), thus enabling all data to be drawn together, and curves (available on request) where each patient could only appear once, either at first attempt at abstinence or first relapse, to confirm the results. Curves were compared using either a log-rank test (for one measurement per patient) or a Cox model with multiple events per subject (frailty model [36, 37]) with or without adjustment for age, gender, severity according to DSM-IV, duration of alcohol misuse, psychiatric comorbidities, treatments and consultation frequency (variable corresponding to the number of consultations divided by duration of follow-up).

Data analysis and graphic results were performed using the R statistical package and its "survival" library . All statistical tests used a two-sided α risk of 5%. A Mann and Whitney test and Fisher's exact test were used to compare means and percentages, respectively. Other tests performed (Cox, log-rank) are stated in the text.

## Competing interests

The author(s) declare that they have no competing interest.

## Authors' contributions

LM and MR designed the study. LM and BF analysed the data. LM, PML and BF drafted the paper. PML and MR supervised and coordinated the research.
